# The Medical Profession and American Slavery

**Published:** 1853-04

**Authors:** 


*The Medical profession and American Slavery.*—A certatin Benjamin W. Richardson, in a letter to the editor of the *Lancet*, (Dec. 4, 1852,) writes as follows:

"I see in the *Times* of to-day, that a meeting of ladies has lately been held at Strafford House, for the purpose of addressing a memorial from the women of England to the women of the United States, in favor of the abolition of slavery.

"While I earnestly admire the philanthropic motives which called these ladies together, and anticipate great good from their labors, I cannot but think that they have set before the English members of the profession of medicine, an example well worthy of being followed."

He then inquires: "Now might not we, the medical men of this country, do much good by transmitting a fitting memorial to our Transatlantic brethren, entreating them to lend their aid to every endeavor that shall be made to abolish slavery?" and he makes a long appeal, characterized by the usual cant and twaddle, that they should do so, very thriftily observing "that the memorial might be prepared at the merest outlay of labor *and at little pecuniary cost.*"

We might suppose, from the grievous complaints continually made in the British Journals, of the evils which afflict the profession in that country, and which have driven, we are told, some members of it to enroll themselves as policemen, and others to advertise for menial situations, even that of gentleman's valet, that the profession had enough to gratify their philanthropy in effecting reforms at home without adding to their ladors by intermeddling with the concerns of those at a distance. When they shall have succeeded in accomplishing a reform in their own condition, in ameliorating that of their laboring population, and in liberating their vast East Indian population from their worse than negro bondage, their example will effect more in stimulating the profession in this country to engage in similar philanthropic enterprises than any memorial they can now send us.—*Med. News avd Lib.*

